# Molecular Biological Determination of *HER2* Status Using Both DNA and RNA Approaches: A Concordance Study with IHC Assessment

**DOI:** 10.3390/ijms26052148

**Published:** 2025-02-27

**Authors:** Ema Ruszova, Ziad Khaznadar, Zuzana Spurkova, Katerina Vlcanova

**Affiliations:** 1 Laboratory of Clinical Genetics, University Hospital Bulovka, 180 81 Prague, Czech Republic; 2 The Central Laboratories, University Hospital Bulovka, 180 81 Prague, Czech Republic; ziad.khaznadar@bulovka.cz; 3 Third Faculty of Medicine, Charles University, 100 00 Prague, Czech Republic; 4 Department of Anatomical Pathology, University Hospital Bulovka, 180 81 Prague, Czech Republic; zuzana.spurkova@bulovka.cz

**Keywords:** HER2/ERBB2, quantitative PCR assessment, gene expression, gene copy number

## Abstract

The immunohistochemical (IHC) or fluorescence/chromogenic in situ hybridization (FISH/CISH) assays for assessing HER2 are now recommended by the American Society of Clinical Oncologists and the College of American Pathologists, but there are an increasing number of published studies describing alternative diagnoses at the molecular level. Inspired by these studies, we established a laboratory-developed test (LDT) to analyze *HER2* status not only at the gene expression level but also at the gene copy number. A precise copy number calculation was fulfilled including the Control Genomic DNA of known concentration, which allowed subsequent assay validation at the DNA level. The results were reported according to the concordant results of the DNA and RNA approaches. By comparing with IHC determination, completely identical results were found in ten blank samples, which underlines the legitimacy of molecular biological approaches in this diagnostic field. An equivocal sample that was positive by IHC and qPCR was found to be negative by the FISH and so it may change the choice of personalized medicine. The topic of this short communication will hopefully contribute to allowing IVD-certified diagnostics based on the *HER2* gene expression profile or copy number to be tested in the Czech Republic as well.

## 1. Introduction

The human gene for epidermal growth factor 2 (*HER2*) [also known as *ERBB2* or epidermal growth factor receptor (*EGFR)2*] encodes a 185 kDa transmembrane glycoprotein with tyrosine kinase activity. Clinically, *HER2* overexpression, which is observed in 10–35% of all breast cancers, has been correlated with serious prognostic breast cancer characteristics, as its amplification corresponds with tumor size, lymph node metastasis, a high S-phase fraction, aneuploidy, and a low level of steroid hormone receptors [1,2].

Recent studies show a strong correlation between *HER2* gene amplification and tamoxifen resistance [3,4]. Anti-HER2 therapy consists of the administration of the monoclonal antibody, e.g., trastuzumab (Herceptin), which is effective in the case of metastasis and/or in combination with chemotherapy [5,6,7].

According to the guidelines of the American Society of Clinical Oncology/College of American Pathologists (ASCO/CAP), there are now established techniques for determining HER2 status in primary tumors based on immunohistochemistry (IHC) or fluorescence/chromogenic in situ hybridization analysis (FISH/CISH) [8]. Nevertheless, there is increasing evidence of equivocal samples with a negative FISH result, which are therefore not eligible for anti-HER2 therapies. Initial estimates put the number of such cases at around 8% [9].

Several papers support the use of molecular biological methods in this area of clinical diagnostics. The authors Wang et al., 2013 reported a 0.82 Spearmen rank correlation (*p* < 0.0001) between qPCR and FISH [10]. Similarly, Nakanishi et al., 2004 stated that real-time PCR provided more objective and quantitative data on *HER2* status and FISH analysis might not show positive signals of lower gene amplification [11]. Thirdly, *HER2-*low BC (breast cancer) samples showed a higher reliability of technique at the molecular level [12] and Gheni et al., 2020 published supportively that the concordance rate between their IHC and qPCR data was 94.4% [1].

The aim of the study is to introduce the determination of *HER2* status using molecular biology methods, both at the level of the number of copies of the *HER2* gene and at the level of its gene expression so that we can evaluate with the advantage of two levels (both DNA and cDNA), which will help us to read particularly ambiguous and doubtful cases accurately.

## 2. Results

### Assessment of HER2 Status

We developed a protocol to determine the copy number of the *HER2* gene by quantitative RT-PCR/qPCR using SYBR green dye I, with which we examined 10 blind DNA samples from the tumor tissue of breast cancer patients. The content of the target DNA in tumor samples was quantified by using standard curves according to the Control Genomic Human DNA (ref.no. 4,312,660 Thermo Fisher Scientific, Waltham, MA, USA) and the same PCR protocol as for gene expression profiling (written in Section 4.3). The *HER2* copy number in each sample was normalized on the basis of its content of the “backbone” reference gene that is located at *21q21*, which has not been found to exhibit alterations in breast cancer patients [5] and its amplification efficiency was found to be similar to the *HER2* gene [13]. Standard curves for both the targets, the *HER2* and the *APP* reference gene, for each run were constructed using threefold serial dilution ranging from 1 ng/µL to 50 pg/µL of the Control DNA (cat.no. 4312660, Thermo Fisher Scientific, Waltham, MA, USA) and CFX Manager Dx software version 3.1 (Bio-Rad, Hercules, CA, USA). Supplementary Figure S2 contains such runs with constructed calibration curves, then the *HER2* copy number was calculated as an *HER2* (ng/μL) to *APP* (ng/μL) ratio. To test the reproducibility, we analyzed samples in duplicates. The amplification of *HER2* was defined as five copies of this gene above the average ploidy of the tumor sample [14]. However, as we found small differences between *APP* and *HER2* amplification efficiency, the threshold for a positive result was appropriately shifted. Unlike the DNA level, no differences in PCR efficiency were discovered at the cDNA level (as seen in Supplementary Figure S1a,b for detailed information).

**Figure 1:**
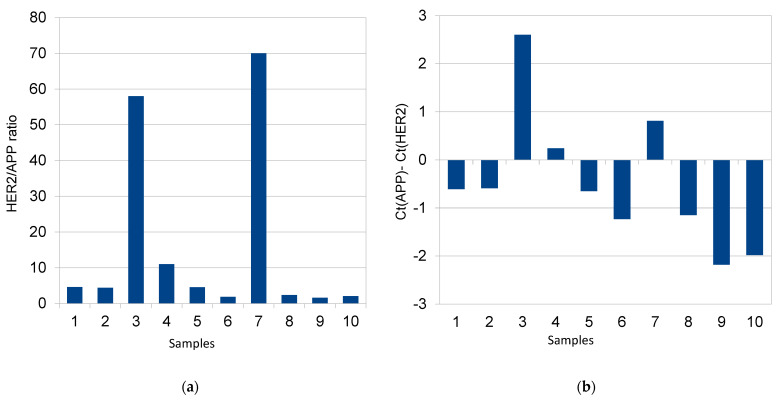
Results of HER2 assessment on both levels, copy number (**a**) and gene expression (**b**), at 10 blind samples. (**a**) Concentration data for *HER2* and *APP* gene dose from FFPE samples. Outputs are normalized according the calibration curves computed for each bio-marker (see Supplementary Figures S2 and S3). Different efficiency of PCR reaction (for targets *HER2* and *APP*) is the reason for considering the cut-off value a bit higher than already published value 2.5×. (**b**) Changes in *HER2* gene expression level in tumoral tissues are normalized to *APP* (reference gene) in consistency with ΔCt method. Comparative Cycle Threshold (Ct) method uses the following formula: Δ*Ct: average reference* (*APP*) *transcript Ct − average target* (*HER2*) *transcript Ct.*

The relative quantification of *HER2* expression was performed using the ΔCt method. For *HER2*-positive samples, the increase in the difference between the Ct values of *HER2* and *APP* transcripts in tumor tissue essentially corresponded to the overexpression of the *HER2* gene (Figure 1b). The real-time PCR gene expression data for another positive sample (no. 7) are shown in Supplementary Figure S3.

**Figure 2:**
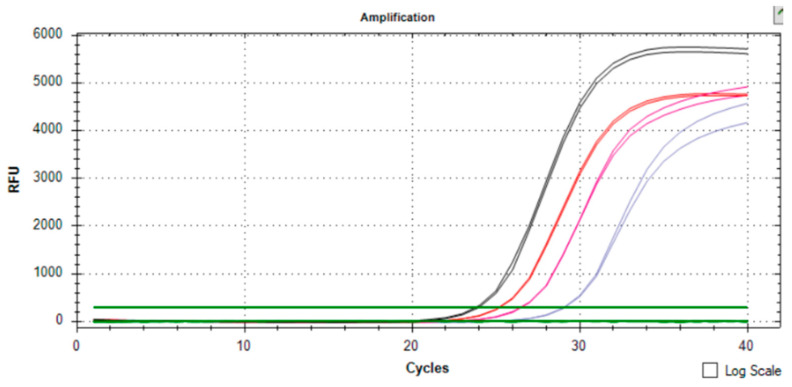
Copy number and HER2 gene expression simultaneous assessment in sample 3. Red line—*HER2* copy number, purple line—*APP* copy number, black line—*HER2* gene expression, pink line—*APP* gene expression.

Samples 3 and 7 were strongly positive (in agreement with the IHC assessment of 3+) and sample no. 4 appeared slightly above the cut-off value (Figure 1a). The dual concept for evaluation also revealed concordant results on the gene expression level for all examined samples (Figure 1b). Figure 2 then shows the simultaneous evaluation of the copy number and gene expression curves in sample no. 3.

## 3. Discussion

Different studies demonstrate that quantitative real-time PCR approaches are valuable tools for the assessment of *HER2* gene overexpression and can provide a reliable alternative to FISH and IHC determinations [15,16,17]. Generally, PCR-based assays can detect changes in the *HER2* gene number as well as gene expression differences. Q-PCR assays based on DNA/cDNA analyses are described as sensitive enough, faster, easy to perform, specific and cost-effective to measure *HER2* alterations and able to analyze multiple samples simultaneously [18]. The high sensitivity of real-time qPCR means that even minute amounts of DNA or RNA can be detected in FFPE tissue [19], opening up the possibility of performing retrospective clinical and molecular studies on the large sample archives stored in pathology institutes [20]. There is evidence from another study that illustrates that determined gene expression ratios are also useful in *HER2*-low breast cancer patients [21].

Nevertheless, qPCR, like any method, has its methodological limitations. One of these is DNA damage and conformational changes in the structure, which can lead to the mis-annealing of primer/probe mixtures [22]. These difficulties can at least be circumvented by heating the template to a high temperature before reaction set-up or by treating the nucleic acids with a cocktail of repair enzymes (e.g., FFPE repair mix, New England BioLab., Ipswich, MA, USA) prior to amplification [23]. Moreover, assay design and the use of random primers in reverse transcription appear to be critical for both long-term archived FFPE blocks and the accurate calculation of differences between focused bio-markers [24]. The presence of PCR inhibitors can be another source of inconvenience when working with FFPE samples. The removal of PCR inhibitors using PCR inhibitor removal columns (e.g., OneStep PCR Inhibitor Removal Kit, ZymoResearch, Irvine, CA, USA) and the accurate design of primer/probe sets allowing the amplification of very short amplicons are ways to overcome these obstacles [25].

To summarize, strongly positive samples from IHC assessments (sample nos. 3 and 7) were also positive using molecular method determination, consistently at both DNA and RNA levels. Regarding the requirements for the content of tumor tissue, usually it is about 20% [26]. Our study seems to be in agreement with another study [9] that molecular approaches are rather sensitive and less demanding on the percentage of tumor cells, with fractions of amplified cells as small as 5%. Our system offers a decisive advantage, the possibility to perform a double evaluation, which facilitates the interpretation of the results and enhances result interpretation flexibility and quality control. In addition, linkage to reference genes enables the *HER2* status to be monitored in a time course, and therefore, it can affect therapy efficiency.

Three samples that were classified as positive by real-time PCR were also positive in the IHC detection. This indicates that real-time PCR does not give false positive results, which is consistent with a previously published study [27]. Conversely, two samples (nos. 4 and 8) positive by the IHC were finally reported as negative by the confirmatory FISH assessments. We came to the consensual conclusion that the *LDT*-assay developed by our laboratory shows better analytical performance than FISH (also detectable in the current study) and would be useful also in patients with *HER2*-low breast cancer (sample no. 4), so that more patients could benefit from anti-HER2 treatment. The consideration here is that the combination of the recommended IHC and FISH/CISH methods still leads to results that fall within the equivocal range and could exclude true positive samples and that qPCR methods could be useful as a complement.

## 4. Materials and Methods

We examined samples of formalin-fixed, paraffin-embedded (FFPE) primary breast cancer tumors obtained from the Pathological Anatomy Department of Bulovka College Hospital (FNB). The samples were routine surgical specimens that were fixed in formalin, processed, and stored according to standard histologic protocols and the rules of the local medical ethics committee of the FNB. The same samples for determination by the recommended IHC technique were evaluated in parallel using quantitative PCR.

### 4.1. DNA/RNA Preparation

On the original slides of each of the samples, a representative area containing tumor cells was marked by a pathologist (Dr. Z. Špurkova). Thereafter, 3–5 microtome slices (diameter 5 µM) were obtained from the corresponding area in each paraffin block. Deparaffinization was performed according to the Agilent Protocol (FFPE extraction procedure, Cat. No. 400925) and RNA/DNA was isolated using CE-IVD-certified diagnostics: the FFPE RNA Purification Kit Dx (Norgen Biotek, Thorold, Ontario, CA, USA) or AmoyDx FFPE DNA/RNA kit (Amoy, Xiamen, China). The clear advantage of the AmoyDx kit was the simultaneous isolation of both RNA and DNA without affecting RNA yields compared to the Norgen Biotek extraction. The concentration of the DNA/RNA was determined using a Qubit 4 Fluorometer (Thermo Fisher Scientific, Waltham, MA, USA). The Quant-iT^TM^ dsDNA HS Assay Kit and Quant -iT^TM^ RNA Assay Kit (Thermo Fisher Scientific, Waltham, MA, USA) for DNA and RNA, respectively, were used for the fluorometric measurement of nucleic acid concentrations.

### 4.2. Targets

Real-time PCR assays for the target gene *HER2* and the reference gene *APP* (encoding the amyloid precursor protein) were run at both DNA and cDNA. The theoretical premise for creating an assay at both levels was the knowledge that *HER2* overexpression was present in all tumors with *HER2* gene amplification but was uncommon in breast cancers without gene amplification [19]. The *APP* gene was selected as a “housekeeping”/“backbone” marker. The design of primers for the gene expression study was performed in ExonSurfer web-tool, allowing us to avoid the amplification of the genomic template (Table 1) [28].

### 4.3. Reverse Transcription and Real-Time PCR of cDNA (Gene Expression Level)

cDNA was synthesized using ProtoScript II First Strand cDNA Synthesis Kit (New England BioLab., Ipswich, MA, USA) according to the manufacturer’s instruction. The Random Primer Mix yields shorter cDNAs on average and can be used for the detection of multiple short RT-PCR products, appropriately designed for fragmented nucleic acids.

The 20 µL reaction mixture contained 2x Luna^®^ Universal qPCR Master Mix (New England Bio Lab., Ipswich, MA, USA), 0.05 µM forward primer, 0.05 µM reverse primer (all from Generi-Biotech s.r.o., H. Kralove, Czech Republic) and 2.5 µL DNA or cDNA. See the New England Biolabs for the exact protocol [29]. In general, the volume of cDNA product should not exceed 1/10 of the PCR reaction volume. The PCR program started with one cycle at 95 °C for 2 min. The amplification was run for 40 cycles with short denaturation at 95 °C for 15 s and annealing with extension at 60 °C for 45 s. All reactions were performed in a Connect CFX real-time PCR system (Bio-Rad, Hercules, CA, USA). Proofs of reactions’ specificity are demonstrated in Supplementary Figure S1c,d, which can be consulted to follow the logic of the procedure.

### 4.4. IHC Assessment

A total of 10 breast cancer tissue samples being recently processed were analyzed immunohistochemically using the c-erbB-2 oncoprotein antibody (DAKO, Glostrup, Denmark) with internal (on slide) positive and negative controls. Staining was performed in a Ventana immunohistochemistry staining system.

Immunostaining was evaluated by a pathologist and was performed according to the ASCO guidelines [30]: negative for 0 (no or weak incomplete staining <10% tumor cells) and 1+ (faint or barely perceptible incomplete membrane staining >10% tumor cells); equivocal for 2+ (weak to moderate complete membrane staining >10% tumor cells); and positive for 3+ (>10% tumor cells with circumferential complete, intense membrane staining). The samples were then sent to the reference laboratory of the Institute of Pathology VFN Prague (Prague, Czech Republic), where the determination was performed using the certified Ventana HER/Neu kit, clone 4B5. Some samples with a negative and indeterminate result (a score of 0+, 1+, or 2+) were subsequently examined by FISH, whereby no amplification of the HER2 gene could be detected (Table 2). FISH was not performed on definitely positive tumors (a score of 3+) (sample nos. 3 and 7).

## 5. Conclusions

While IHC requires knowledge of how to read results in order to achieve reproducibility and may replace established techniques, it could remove the need to use expensive antibodies and reagents; on the contrary, molecular–biological approaches could be considered where large numbers of patients are not expected. We anticipate their use especially in small laboratories without the necessary equipment (a fluorescent microscope), where the risk of overexposure to chemistry increases and where huge manual workloads may compromise the quality of results.

The increasing number of publications and new diagnostics (e.g., MammaTyper^®^, OncotypeDX^®^ test, Xpert^®^ Breast Cancer STRAT4) on the successful implementation of *HER2* determination by molecular biology methods speaks more and more in favor of the idea that it is not the inherent limitations of the method but its good standardization that is important for its complementary use as a third alternative.

## Figures and Tables

**Table 1:** Design of primers and probes used in quantitative real-time PCR.

Product Size/* Source	Sequence	Oligonucleotide	Gene	Level
80 bp	5′-GT CCT GGA AGC CAC AAG G-3′	Fwd	*HER2*	gDNA
* [5]	5′-GGT TTT CCC ACC ACA TCC TCT-3′	Rw	*HER2*	gDNA
72 bp	5′-TTT GTG TGC TCT CCC AGG TCT-3′	Fwd	*APP*	gDNA
* [5]	5′-TGG TCA CTG GTT GGT TGG C-3′	Rw	*APP*	gDNA
86 bp	5′-AGACACGTTTGAGTCCATGC-3′	Fwd	*HER2*	cDNA
* ExonSurfer	5′-AAAGGTAGTTGTAGGGACAGGC-3′	Rw	*HER2*	cDNA
124 bp	5′-TTGTCTTCACTCCCATCTGC-3’	Fwd	*APP*	cDNA
* ExonSurfer	5′-AGTTTGTGTGTTGCCCACTG-3’	Rw	*APP*	cDNA

**Table 2:** IHC and FISH assessment results.

Sample No.	Sex/Age	Tumor Subtype	IHC; Semi-Quantitative Scale	FISH-Amplification; Qualitative Scale
1	F/76	Luminal A	1	-
2	F/79	Luminal A	0	-
3	F/45	Luminal B, Her2 positive	3	-
4	F/76	Luminal B, Her2 negative	2	unproven
5	F/74	Luminal B, Her2 negative	0	-
6	F/71	Luminal B, Her2 negative	0	-
7	F/51	Her2-positive (non-luminal)	3	-
8	F/28	Triple negative	1	unproven
9	F/54	Triple negative	0	unproven
10	F/72	Triple negative	0	unproven

## Data Availability

The original contributions presented in the study are included in the article. Further inquiries can be directed to the corresponding author/s.

## Supplementary Materials

The supporting information can be downloaded at: https://www.mdpi.com/article/10.3390/ijms26052148/s1.
