# Supplementary material for: Molecular Biological Determination of HER2 Status Using Both DNA and RNA Approaches: A Concordance Study with IHC Assessment
Source: Int J Mol Sci. 2025 Feb 27;26(5):2148. doi: 10.3390/ijms26052148 (PMC11899855; doi:10.3390/ijms26052148)

SUPPLEMENTARY FIGURES

Supplementary Figure S1:

(a) qPCR of ***APP*** gene (orange) and ***HER2*** gene (blue) using male Human Genomic DNA standard as a template (cat.no. 4312660, ThermoFisher, 1ng/µl, 100pg/µl, 50pg/µl) showing differences between amplification efficiency.

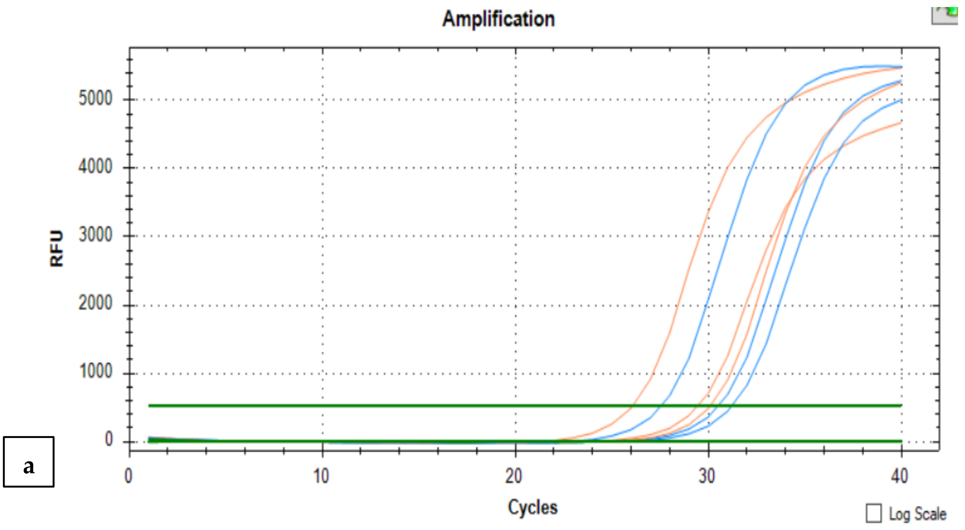

a

(b) qPCR of ***APP*** transcripts (red, detectable transcripts: ENST00000359726; ENST00000463070; ENST00000474136;ENST00000448850;ENST00000358918;ENST00000439274;ENST00000440126;ENST00000357903;ENST00000348990;ENST00000354192;ENST00000346798) and ***HER2*** transcripts (green, detectable transcripts: ENST00000541774; ENST00000406381; ENST00000578502; ENST00000583038;ENST00000584908;ENST00000269571;ENST00000578373;ENST00000584450;ENST00000578199;ENST00000584601); using pooled cDNA sample as a template; no differences in efficacy were shown.

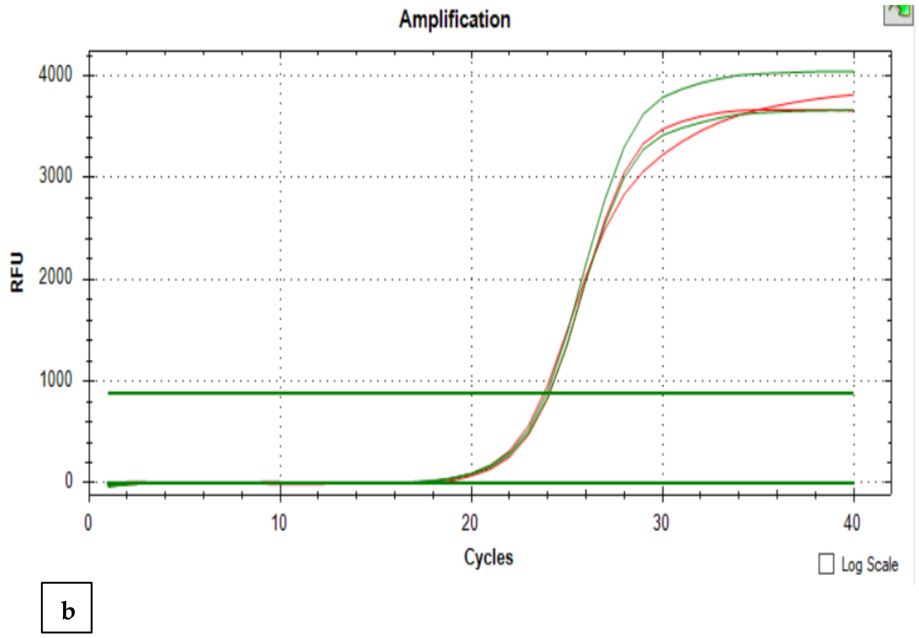

b

SUPPLEMENTARY FIGURES

(c) High resolution melting profiles of ***APP*** (orange) and ***HER2*** gene (blue). The specificity of the PCR reactions was demonstrated at the DNA level.

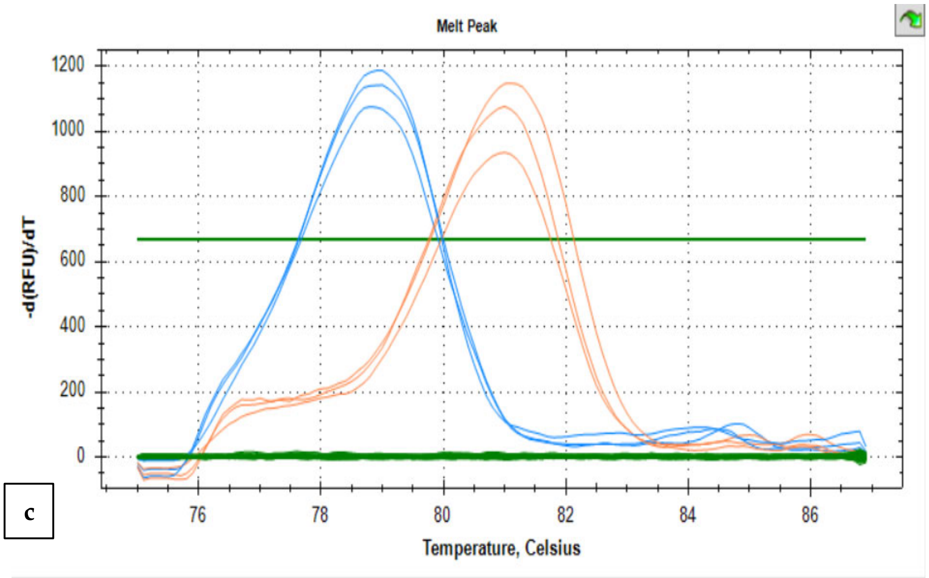

c

(d) High resolution melting profiles of ***APP*** (red) and ***HER2*** gene (green). The specificity of the PCR reactions was demonstrated at the cDNA level.

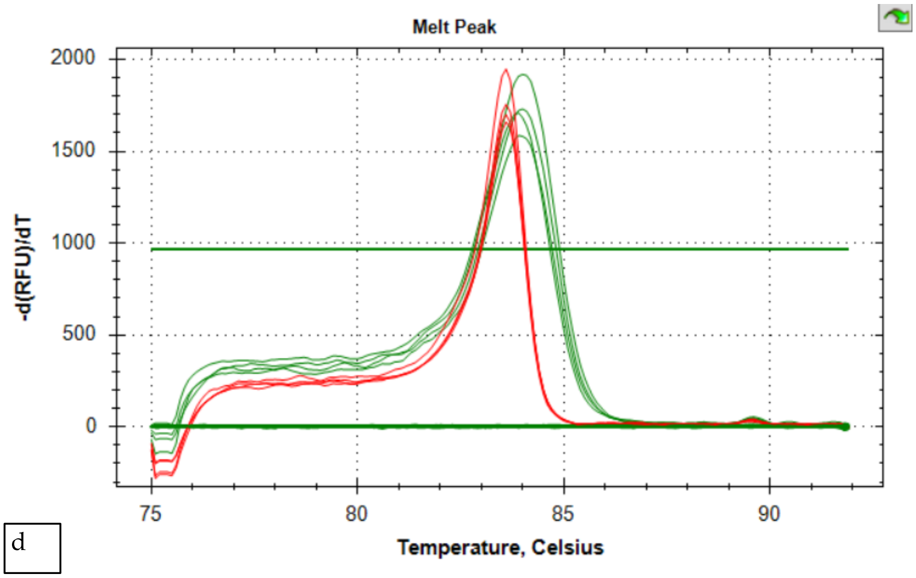

d

Supplementary Figure S2: CNV analysis of positive **sample no. 3**. Q-PCR of ***HER2*** gene (red), ***APP*** gene (**black)** and their absolute quantification using the Human Genomic DNA Standard (cat.no. 4312660, Thermo

SUPPLEMENTARY FIGURES

Fisher, 1ng/µl, 100ng/µl, 50pg/µl). Calibration curves created for both genes: ***APP*** gene (orange); ***HER2*** gene (blue).

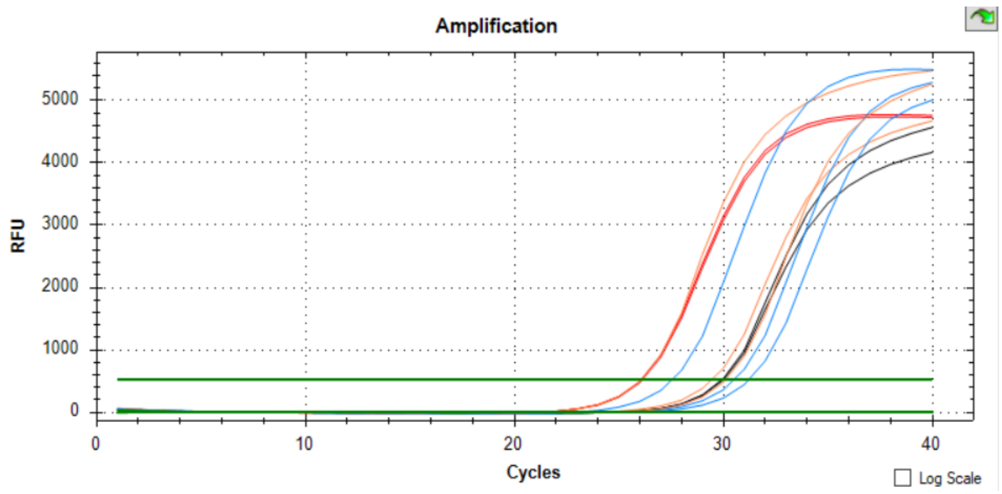

Supplementary Figure S3: **Sample no. 7**- QPCR test for HER2 gene copy number and its gene expression, simultaneous assessment. Absolute quantification of DNA using calibration curves generated with the male Human Genomic DNA Standard (cat.no. 4312660, Thermo Fisher, green—1ng/µL, 100 pg/µL, 50 pg/µL; **APP** gene (orange); ***HER2*** gene (blue)). Q-PCR of the DNA template for ***APP*** gene (green) and ***HER2*** gene (red). Q-RT-PCR of the cDNA template for ***APP*** transcripts (brown) and ***HER2*** transcripts (purple).

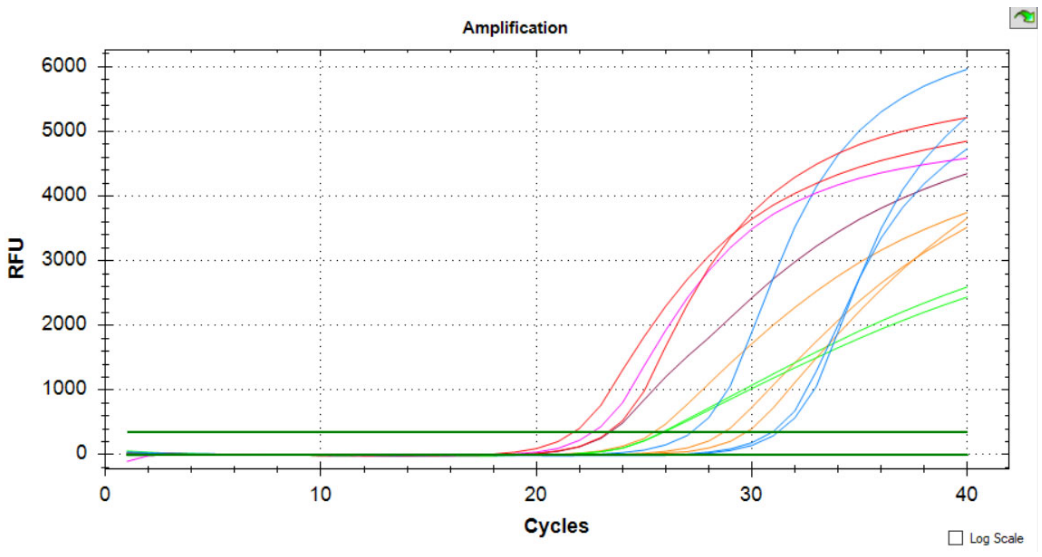

Supplement: Supplementary file 1 [file ijms-26-02148-s001.zip › ijms-3330211-supplementary.pdf]
